# Exposure to environmental stressors result in increased viral load and further reduction of production parameters in pigs experimentally infected with PCV2b

**DOI:** 10.1016/j.vetmic.2015.03.010

**Published:** 2015-06-12

**Authors:** Robert Patterson, Amanda Nevel, Adriana V. Diaz, Henny M. Martineau, Theo Demmers, Christopher Browne, Bettina Mavrommatis, Dirk Werling

**Affiliations:** a Royal Veterinary College, Department of Pathology and Pathogen Biology, Hawkshead Lane, AL9 7TA, UK; b Royal Veterinary College, Department of Clinical Sciences, Hawkshead Lane, AL9 7TA, UK

**Keywords:** PCV2, PCVAD, Environment, Housing, Temperature, qPCR, ADG, FCR

## Abstract

Porcine circovirus type 2 (PCV2) has been identified as the essential, but not sole, underlying infectious component for PCV-associated diseases (PCVAD). Several co-factors have been suggested to convert an infection with PCV2 into the clinical signs of PCVAD, including co-infection with a secondary pathogen and the genetic background of the pig. In the present study, we investigated the role of environmental stressors in the form of changes in environmental temperature and increased stocking-density on viral load in serum and tissue, average daily weight gain (ADG) and food conversion rate (FCR) of pigs experimentally infected with a defined PCV2b strain over an eight week period. These stressors were identified recently as risk factors leading to the occurrence of severe PCVAD on a farm level. In the current study, PCV2-free pigs were housed in separate, environmentally controlled rooms, and the experiment was performed in a 2 × 2 factorial design. In general, PCV2b infection reduced ADG and increased FCR, and these were further impacted on by the environmental stressors. Furthermore, all stressors led to an increased viral load in serum and tissue as assessed by qPCR, although levels did not reach statistical significance. Our data suggest that there is no need for an additional pathogen to develop PCVAD in conventional status pigs, and growth retardation and clinical signs can be induced in PCV2 infected pigs that are exposed to environmental stressors alone.

## Highlights

• Environmental stress increases viral load of PCV2b in serum and tissue.
• Environmental stress exacerbates PCV2b induced weight loss.
• Environmental stress and PCV2b reduce ADG and impact negatively on FCR.

## 1 Introduction

Porcine circovirus 2 (PCV2) is a non-enveloped single-stranded DNA virus with a diameter of less than 20 nm that was isolated from diseased pigs in the late 1990s (Allan et al., 1998; Mankertz, 2012). The virus has a small genome of less than 2000 nucleotides compromising three open reading frames. Thus far, there are three reported genotypes of PCV2. These are PCV2a, PCV2b and PCV2c, each of which have several subtypes. In the UK the dominant strain circulating on severely infected farms belongs to the PCV2b genotype as determined by a recent study (Wieland et al., 2012). In the early days of PCV2 occurrence, the virus was associated with post-weaning multi-systemic wasting syndrome (PMWS) in pigs, which had a major impact on the swine industry primarily due to high post-weaning mortality rates on affected units. Pigs that develop PMWS usually present with a range of clinical signs including diarrhoea, jaundice, respiratory distress, wasting and enlarged lymph nodes. Pigs most commonly at risk are between eight and fifteen weeks of age, which is due to a rapid decline of maternally derived antibodies in the serum (Lukert, 1999). As the disease progresses, mortality declines and subsequent production losses are due to reduced growth rates, increased feed conversion ratios and deaths in post-weaned pigs through subsequent secondary infections (Alarcon et al., 2011b). For these reasons, the nomenclature has moved away from PMWS and it is now more often termed PCV-associated disease (PCVAD) (Opriessnig and Langohr, 2013). Control of PCVAD has presented many challenges due to its nature as multi-factorial disease. Current control strategies include implementation of an “all in-all out” replacement system, as well as high standards of hygiene and biosecurity on the pig farm. However, due to the lack of a viral envelope, PCV2 is notoriously resistant to inactivation by common detergents and disinfectants (Ellis, 2000; Ghebremariam and Gruys, 2005). Vaccination is an additional control strategy and there are currently five commercially available PCV2 vaccines. All of these are against the PCV2a genotype and are either an inactivated whole virus vaccine or subunit vaccine based on the Cap protein, which is the major immunogenic protein of PCV2 (Beach and Meng, 2012). None of the vaccines on the market have been tested in single pathogen controlled challenge experiments due to the lack of an available challenge model. Registration and licensing requirements have been based on either field studies or from co-infected pigs (where at least one other pathogen is used in an artificial challenge model) (Beach and Meng, 2012).

Although PCV2 is the necessary causal agent, many studies have demonstrated that infection with the virus alone is not sufficient to induce clinical signs of PCVAD in pigs; however, there is increasing evidence of subclinical infection (Alarcon et al., 2011b; Allan et al., 1998; Ellis et al., 1998; Nayar et al., 1997). Young et al. (2011) demonstrated that in pig farms with no visible PCVAD, vaccination significantly improved production parameters (Young et al., 2011). PCV2 is considered to be widespread in swine, whereas PCVAD is usually fairly sporadic (Ghebremariam and Gruys, 2005; Larochelle et al., 2003), therefore subclinical PCVAD is likely to be underestimated. Indeed a number of studies have demonstrated that, while PCV2 is detected in both PCVAD-affected and apparently healthy pigs, viral load is higher in PCVAD sufferers (Alarcon et al., 2011b; Brunborg et al., 2004; Grau-Roma et al., 2009; Ladekjaer-Mikkelsen et al., 2002; Sibila et al., 2004) and vaccination of such herds results in a benefit to production indicating that disease is present, but not detected. There has been much speculation and research into the co-factors contributing to the development of PCVAD in PCV2 infected pigs. These include the genotype of the infecting virus with PCV2b having a higher association with disease than PCV2a (Rose et al., 2012) as well as the immune status of the sow and the age at which the piglet is exposed to PCV2 (Grau-Roma et al., 2009). A number of studies have also demonstrated that co-infection of pigs with porcine reproductive and respiratory syndrome virus (PRRSV), porcine parvovirus (PPV) and *Mycoplasma hyopneumoniae* can exacerbate PCV2 infection and can lead to PCVAD. This co-infection has been shown to increase PCV2 replication in the host and also to modify cytokine production and profile (Opriessnig and Halbur, 2012; Segales et al., 2013). Recently, the role of environmental factors and their contribution to the onset of PCVAD has been explored. Housing conditions, hygiene, biosecurity and husbandry have all been linked to PCVAD development (Segales et al., 2013). A recent study demonstrated that reduced pen size and cross-fostering in farrowing crates alter the course of PCV2 infection, favouring earlier infections and therefore possibly exacerbating disease (Andraud et al., 2009). It seems that PCVAD is a truly multifactorial disease and disease progression may not only be dependent on PCV2 infection and one other contributing factor but could depend on a multitude of factors. Currently, little is known about the impact of these different co-factors in the outcome and severity of disease. During their life, pigs are exposed to many environmental stressors in addition to weaning; these include changes in temperature, mixing, noise and shipping. Many of these have been shown to suppress the immune system and therefore increase susceptibility to disease (Kelley, 1980; McGlone et al., 1993). However, whether these environmental stressors affect progression of PCV2 infection is currently poorly understood.

Using recently identified environmental risk-factors for occurrence of PCVAD in a herd (Alarcon et al., 2011a,b), we investigated the role of these potential co-factors on PCV2 infection with the aim of developing a disease model for PCVAD which does not rely on gnotobiotic pigs.

## 2 Material and methods

### 2.1 Ethics statement

All animal studies were performed according to the regulations and guidance provided under the UK Home Office Animals (Scientific Procedures) Act 1986. Experimental protocols were approved under project licence number PPL 70/7219, as well as the RVC Ethics and Welfare Committee.

### 2.2 Animals

In a cross-sectional study of 114 farms in England in 2008, antibodies against PCV2 were detected in 99.1% of herds and PCV2 was detected by PCR on 90.4% of farms, indicating a nearly endemic infection (Wieland et al., 2010). However, we were able to purchase a total of 54 large white × landrace pigs of a similar age from a commercial farm that tested PCV2 free as by PCR and antibody ELISA before study recruitment. Pigs were randomly allocated to nine groups (*n* = 6). Eight of the nine groups were replicated in a 2 × 2 factorial design to maximise experimental achievement by reducing animal use (Shaw et al., 2002), meaning that a total of 12 animals were tested for each condition, with an additional six pigs in the 9th group which served as a functional control and were not exposed to PCV2 or any environmental stress.

### 2.3 Virus preparation for infection

To avoid potential false-positive results due to LPS contaminations of reagents/media, all substances used were tested for their LPS content using the Endosafe-PTS system (Charles River, Charleston, USA). Samples with endotoxin content below 0.01 EU ml^−1^ were considered as LPS-free.

A previously characterized PCV2b isolate from the UK (GenBank accession number JX193799; (Wieland et al., 2012) was used to generate the virus stock for the experimental infections. The virus was propagated in type-I IFN^KO^ PK15-ALR-NPro cells, free of PCV1 and PCV2. Cells were cultured in MEM containing Earle's salts supplemented with 10% (v/v) tetracycline-free foetal bovine serum (FBS) (Clontech, Saint-Germain-en-Laye, France). To induce the tetracycline-regulated expression of the IFN-KO, tetracycline was added 2 h pre-inoculation. Cell monolayers were inoculated at 50% confluence. After 18 h incubation at 37 °C, the inoculum was removed and retained, and the monolayer treated with 300 mM D-glucosamine in Hanks balanced salt solution (HBSS) for 30 min at 37 °C. After removal of the glucosamine and subsequent washes with HBSS, cultures were overlaid with retained inoculum. Cultures were overlaid with media 24 h post-inoculation. After further three days incubation, media was removed and retained and virus harvested from trypsinised cells by freeze-thawing. The cell lysate was clarified by centrifugation at 1000 × *g* for 5 min at 4 °C and the supernatant added to the retained media. This virus suspension was then concentrated approximately 10-fold using dialysis tubing (Spectra/Por, Biotech Cellulose Ester membrane; Spectrum Europe B.V.) in polyethylene glycol (PEG 12000 flake; Whyte Chemicals Ltd.) at 4 °C. Concentrated virus suspension was subsequently dialysed in MEM overnight and aliquoted. PCV2 stocks were titrated on PK15-ALR-NPro cells as described elsewhere. The titre of the virus stock was determined by qPCR.

### 2.4 Experimental design

In the study design, four treatment groups, one challenged only control group (each with n = 6 animals, repeated in a 2 × 2 study design for a total of 12 animals per treatment in two separate rooms), and one unchallenged control group were allocated to nine identical rooms, all of which had an isolated environmental system, allowing for control of airflow, humidity and temperature. At four weeks of age (Day 0) treatment groups and challenged control groups were inoculated intra-nasally with 1 × 10^10^ PCV2 particles in 5 ml media. Non challenged controls (C) were inoculated with 5 ml of virus free media. One treatment group was inoculated with virus (V) but not subjected to other environmental stressors. The remaining groups, all inoculated with virus, were subjected to either high stocking density (V SD), high environmental temperature (V T) or both, high stocking density and high temperature combined (V SD T). High stocking density was calculated as DEFRA guidelines (https://www.gov.uk/pig-welfare-regulations) minus 25% for the average weight of pig in that group. Areas were altered weekly, after weekly weights were recorded. The three control groups were housed at an optimal stocking rate based on DEFRA guidelines. In high environmental temperature rooms, the air temperature was maintained at 5 °C above upper critical temperature for pigs and was between 30 °C and 35 °C throughout the study. Air temperature was altered weekly according to the size of the pigs. Air temperature for control pigs was maintained at the thermo-neutral zone and was between 20 °C and 25 °C during the study. Throughout this study, two husbandry teams were employed to avoid transfer of virus between treatment and the unchallenged control room. All pigs were observed daily for signs of disease. Feed intake for each pen was recorded daily and pigs were weighed weekly. Blood samples were collected weekly and serum was stored at −80 °C until analysis. On day 56 pigs were humanely killed by captive bolt stunning, pithing and bleeding. Necropsies were performed and samples of mesenteric lymph node, inguinal lymph node, lung and bone marrow were collected and stored at −20 °C for later analysis of PCV2 load by qPCR.

### 2.5 Measurement of weight and calculation of feed conversion ratios

Each pig was weighed at weekly intervals. Additionally, mass of feed consumed in kg was determined weekly for each room. Food conversion ratios (FCR) were calculated by dividing the mass of feed consumed per week, per room, by the sum of weight gains per room for the corresponding week interval.

### 2.6 Isolation of total DNA from tissue and serum samples

Total DNA was isolated from between 25 and 50 mg of tissue sample collected from all pigs at post-mortem using a DNeasy blood and tissue kit (Qiagen) according to the manufacturer's instructions. Isolated DNA was eluted in sterile and nucleic acid-free water (Sigma-Aldrich) and the DNA concentration was determined using an Infinite 200 Nanoquant spectrophotometer (Tecan). Eluted DNA was stored at −20 °C. In addition, DNA was isolated from 200 μl cell-free serum collected from pigs weekly in one room of each treatment during the course of the study. The QiAMPMinElute Virus spin kit (Qiagen) was used and the manufacturer's instructions were followed. Isolated DNA was eluted in sterile and nucleic acid-free water (Sigma-Aldrich) and the DNA concentration was determined using an Infinite 200 Nanoquant spectrophotometer (Tecan). Eluted DNA was stored at −20 °C.

### 2.7 Quantification of PCV2 in tissue or serum samples

The number of PCV2 copies per ng of DNA isolated from each tissue/serum sample was determined by comparison to known standards using qPCR. Each sample was measured in triplicate and in a final volume of 20 μl per well in Microamp Fast Optical 48-microtiter well plates (Applied Biosystems). Each well contained, 2 μl of DNA (standard or test sample), 10 μl of 2 × TaqMan Universal Master Mix II (Applied Biosystems), 50 pmol of each primer (Forward: 5′-GCTCTYTATCGGAGGATTAC-3′, Reverse: 5′-ATAAAAACCATTACGAWGTGATA-3′) (MWG) and 2.5 μM of TaqMan probe (5′FAM-CCATGCCCTGAATTTCCATATGAAAT-3′TAMRA) (Applied Biosystems). The volume of each well was adjusted to 20 μl by addition of nuclease-free water (Sigma-Aldrich). The TaqMan probe and the primers were designed to target a partial (137 bp) sequence of the ORF1 of PCV2 (Grierson et al., 2004). Standard measurements were performed in a 10-fold serial dilution from 10^9^ PCV2 copies to 0 copies. After plate set up, the qPCR was performed in a StepOne Real-time PCR machine (Applied Biosystems) and StepOne software version 2.2.2 (Applied Biosystems). The cycling conditions were 95 °C for 10 min for polymerase activation followed by 40 cycles of denaturation at 95 °C for 15 s and annealing/extension at 55 °C for 1 min. Data were analysed using either StepOne software version 2.2.2 (Applied Biosystems) or Excel 2010 (Microsoft). Ct values of triplicate sample and standard measurements were averaged and this average Ct value was used to calculate the PCV2 ORF1 copy number in samples by comparison to known standards. The copy number in each sample was then divided by the nanogram of DNA in that sample to give the PCV2 copy number per ng of DNA.

### 2.8 Statistical analysis

Average daily weight gain (ADG) values were calculated for each pig by dividing the difference in weights from one week to the previous week by the number of days between both measurements. ADG (gram per day) were analysed through a One Way Analysis of Variance (ANOVA) selecting cases by study week with a Least Square Difference (LSD) Post-hoc, using IBM SPSS statistics 21 software package. A *P*-value <0.05 was considered significant.

The mean copy number of viral DNA found in tissue and serum samples was calculated for each room using GraphPad Prism (Version 6 for Windows). Differences between group means were statistically verified by ANOVA after assessment for normal distribution, followed by a modified *t*-test (Bonferroni) for selected time-points. A *P*-value *<* 0.05 was considered significant.

## 3 Results

Four pigs either died or were euthanized before the end of the study. Of these four pigs, one pig from the V SD group was euthanized at week 5 post inoculation, after veterinary inspection concluded it was showing signs of PCVAD (pyrexia, dehydration, emaciation, lethargy, increased respiratory effort). In addition, one pig from the V T group was found dead the day after weighing and blood sampling at week 4. The pig had evidence of diarrhoea ante-mortem. Post-mortem examination revealed small intestine torsion, possibly secondary to gas build up from the diarrhoea and precipitated by handling for the blood sampling. The other two pigs from the V T group were euthanized for lameness at week 7 post inoculation considering their welfare.

### 3.1 The combination of PCV2b infection and environmental stressors further impacts on growth parameters

**Fig. 1:**
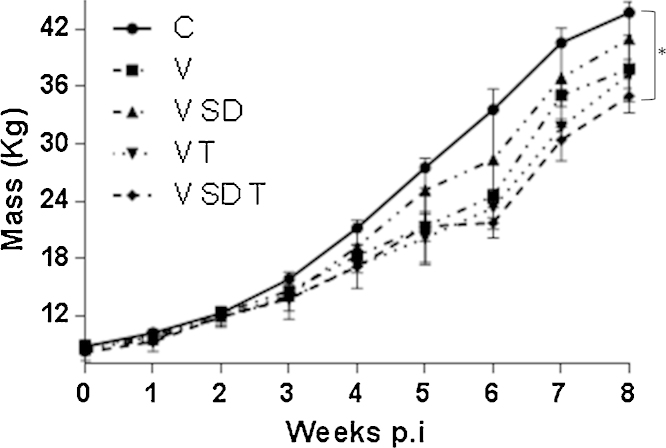
*Mean weekly mass of pigs infected with PCV2b for 8 weeks and subjected to different environmental stresses*. Pigs were infected with PCV2b and subjected to high stocking density (SD), high temperatures (T) or both (SD T). Pigs were weighed weekly and the average weight for the pigs subjected to each condition was recorded. Significant different values were analysed by one-way ANOVA, with ^*^p < 0.05.

**Fig. 2:**
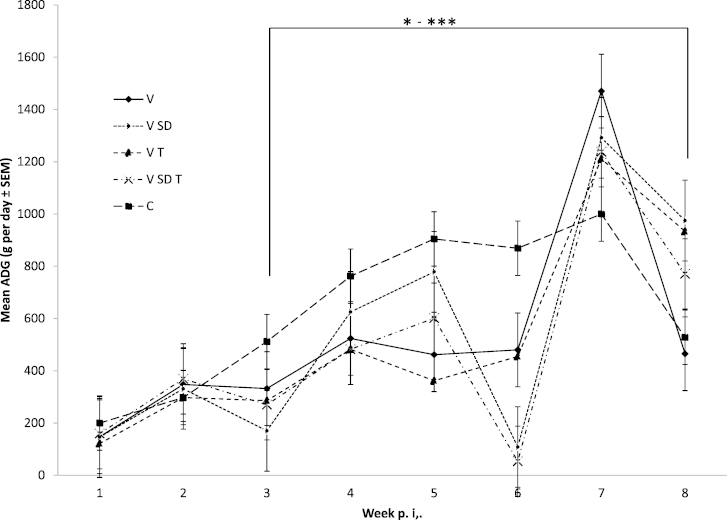
*Mean Average daily weight gain (ADG) of treatment groups per study week*. With the exception of control pigs (C), all pigs were infected with PCV2b (V) on week 0 and subjected to high stocking density (SD), high temperatures (T) or both (SD T). Weights were recorded weekly up to 8 weeks post inoculation. Average daily weight gain per week was calculated for each pig, and a mean ADG per treatment group estimated. Stars shown above the graph indicate significant ANOVA *p*-values between the C and treatment groups, with: ^*^p < 0.05; ^**^p < 0.01; ^***^p < 0.001. *P*-values for LSD post hoc test are presented within the text.

Each pig in the study was weighed weekly and the mass of feed consumed by pigs in each room throughout each week was recorded. The mean weekly mass (Fig. 1) and the mean average daily weight gain (ADG, Fig. 2) and feed conversion ratios (FCR, Table 1) were recorded for each room.

At week 8, the mass (kg) of pigs in the C group (43 kg ± 0.75 kg) compared with pigs in the V SD T group showed a significant difference of 8 kg (35 kg ± 1.25 kg, *p* < 0.05; Fig. 1). Differences between control animals and animals in other groups were just below the level of significance.

There was no statistically significant difference in mean ADG (Fig. 2) between groups as determined by one-way ANOVA at week 1 (*F*(4,49) = 0.929, *p* = 0.455) and week 2 (*F*(4,49) = 0.571, *p* = 0.685) post inoculation.

From week 3 to week 8 post inoculation, the ADG means differed significantly between the groups, as analysed by one-way ANOVA, with *p*-values ranging from *p* < 0.05 to *p* < 0.001. At week 3, the LSD post-hoc test revealed that the ADG was statistically significantly lower in the V group (332.14 ± 194.27 g/day, *p* < 0.05), V SD group (170.23 ± 108.70 g/day, *p* < 0.001), V T group (285.71 ± 95.54 d/day, *p* < 0.01) and the V SD T group (270.24 ± 102.01 g/day, *p* < 0.01) compared with the C group (511.90 ± 29.16 g/day).

At week 4, the LSD post-hoc test revealed that the ADG was statistically significantly lower in the V group (523.81 ± 226.56 g/day, *p*, 0.05), the V T group (482.14 ± 180.51 g/day, *p* < 0.01) and the V SD T group (482.14 ± 175.29 g/day, *p* < 0.01) compared with the C group (761.90 ± 147.54 g/day). As before, there was no significance between the values obtained for the other groups.

At week 5, the LSD post-hoc test revealed that the ADG was statistically significantly lower in the V group (461.58 ± 303.33 g/day, *p* < 0.05), the V T group (389.61 ± 344.58 g/day, *p* < 0.001) and the V SD T group (601.19 ± 159.60 g/day, *p* < 0.05) compared with the C group (904.76 ± 173.01 g/day).

At week 6, the LSD post-hoc test revealed that the ADG was statistically significantly lower in the V group (480.16 ± 372.72 g/day, *p* < 0.05), the V SD group (103.90 ± 187.26 g/day, *p* < 0.001), the V T group (422.08 ± 386.58 g/day, *p* < 0.01) and the V SD T group (53.57 ± 101.59 g/day, *p* = 0.0001) compared with the C group (869.05 ± 70.23 g/day).

At week 7, the LSD post-hoc test revealed that the ADG was statistically significantly higher in V group (1470.24 ± 123.56 g/day, p < 0.001), V SD group (1305.19 ± 209.69 g/day, *p* < 0.01), V T group (1198.41 ± 150.58 g/day, *p* < 0.05) and V SD T group (1238.09 ± 186.10 g/day, *p* < 0.05) compared with the C group (1000.00 ± 135.53 g/day). Until this time-point, no statistically significant differences were seen between V SD and V T group, V SD and V SD T as well as V T and V SD T.

At week 8, the LSD post-hoc test revealed that the ADG was statistically significantly higher in V SD group (962.12 ± 239.69 g/day, *p* < 0.05) and V T group (953.70 ± 273.58 g/day, *p* < 0.05) compared with the C group (527.78 ± 208.61 g/day). Statistically significant differences (*p* < 0.05) were seen between V group (465.28 ± 308.68 g/day) and V SD T group (770.83 ± 261.72 g/day). There were no statistically significant differences between C and V group (*p* = 0.641), C and V SD T group (*p* = 0.075), V SD and V T (*p* = 0.944), V SD and V SD T (*p* = 0.092) and V T and V SD T (*p* = 0.126).

Over the entire study period, C group had the highest ADG (636.06 g/day ± 13.79), followed by the V group (528.79 g/day ± 29.19), the V SD group (518.45 g/day ± 40.12), the V T group (457.08 g/day ± 41.00). Lowest ADG was recorded for the V SD T group (488.03 g/day ± 23.38). Throughout the study, the greatest variation in ADG was observed within the V SD and V T groups, which also had the highest standard error among groups. Moreover, both these groups had pigs, which presented with clinical signs of PCV-systemic disease, which would have affected their ADG and FCR.

Expected FCR for pigs in the UK (Garth Stockmanship Standards) varies from 1.3 at weaning to 2.3 at 12 weeks of age, which coincides with the length of the study. FCR of pigs in the C group was within these ranges, ending the study with a FCR of 2.93, meaning they gained 1 kg of live weight for every 2.93 kg of feed consumed (Table 1 and Table 2). In comparison, inoculated pigs presented a wider variation in FCR during the study, with high to very high FCR at week 5 in a V group room and a V T group room, respectively, and very high FCR for the rest of treatment rooms at week 6 (Table 2). This was mainly due to low weight gain although feed consumed was lower for all treatment rooms compared with control group at week 6 post inoculation. Interestingly at week 5, room 9 (V T) consumed less feed than the control in contrast to room 10 (V) were feed consumption was higher than in the control group (Table 2).

### 3.2 PCV2b viral load in the serum and tissue samples is increased by environmental stressors

**Fig. 3:**
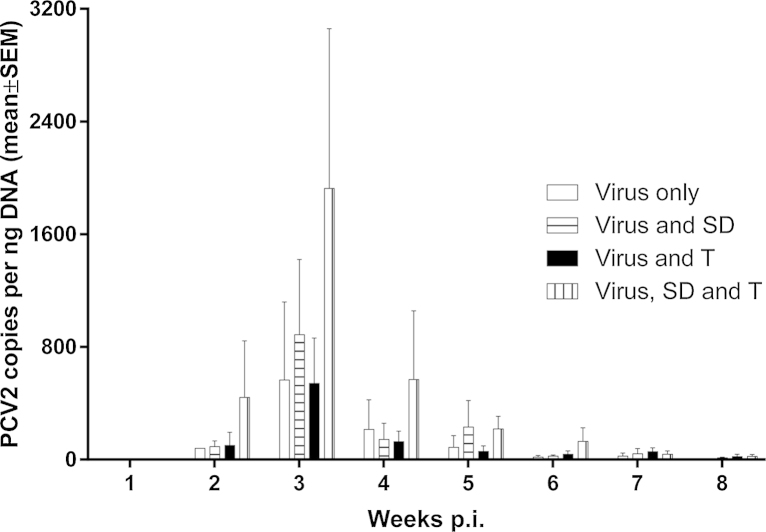
*Viral copies in serum samples taken from pigs infected with PCV2b and subjected to different environmental stresses*. Pigs were infected with PCV2b (V) and subjected to high stocking density (V SD), high temperatures (V T) or both (V SD T). Serum samples were taken weekly up to 8-weeks post-infection and tested for viral load by qPCR. Values are expressed as mean ± standard error of the mean (SEM) PCV2 copies numbers per ng DNA.

**Fig. 4:**
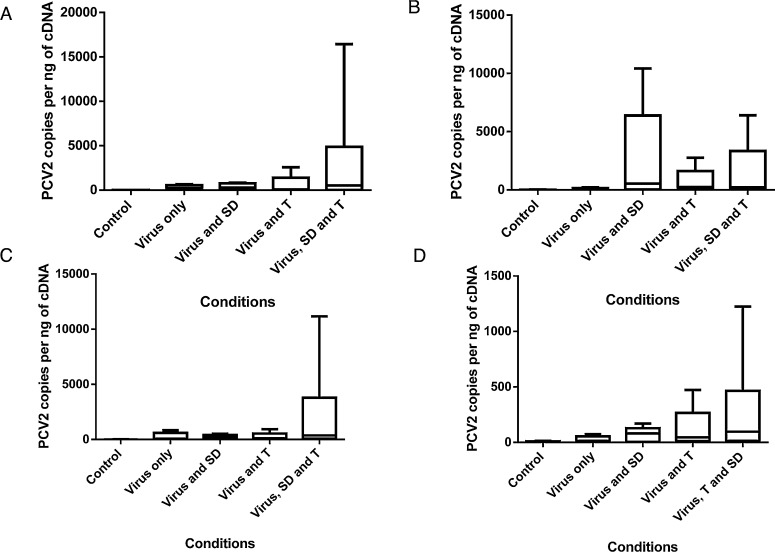
Viral copies in mesenteric lymph node (A), inguinal lymph node (B), lung (C) and bone marrow (D) taken from pigs infected with PCV2b for 8 weeks and subjected to different environmental stresses. Pigs were infected with PCV2b (V) and subjected to high stocking density (V SD), high temperatures (V T) or both (V SD T). Tissue samples were collected post-mortem at 8-weeks post-infection and tested for viral load by qPCR. Values are expressed as mean ± standard error of the mean (SEM) PCV2 copies numbers per ng DNA.

To assess whether exposure of pigs to environmental stress would impact on the viral load detected, in addition to the weight differences seen, serum (Fig. 3) and tissues samples (Fig. 4) were analysed by qPCR for PCV2 DNA. All pigs were serum PCV2b negative at week 1 post-infection and uninfected controls remained serum PCV2b negative throughout the eight-week study (Fig. 3). Viraemia in PCV2b-infected pigs always peaked at three weeks post infection and then dropped rapidly up to eight weeks post infection regardless of which environmental stressors were also applied. Interestingly, a much higher peak in serum viraemia was detected in V SD T pigs compared with V SD or V T pigs (Fig. 3), but the differences did not reach statistical significance due to large variation between pigs in the same group. These results indicate that exposure to environmental stresses potentially increases the viral load in the serum of PCV2 infected pigs.

In addition to serum samples, the amount of PCV2 DNA in samples obtained from mesenteric lymph nodes, inguinal lymph nodes, lung and bone marrow were analysed. All samples were collected post-mortem at eight weeks post infection, and viral DNA was analysed as described. A comparison between the different tissue samples showed that the highest viral load was observed in the mesenteric lymph node (Fig. 4A), followed by the inguinal lymph node (Fig. 4B), lung (Fig. 4C) and bone marrow (Fig. 4D). Independent of the tissue tested, there was a tendency that viral load was highest in samples obtained from pigs exposed to all conditions (V SD T), with the exception of the samples taken from the inguinal lymph node. Furthermore, there was a tendency that samples taken from infected pigs exposed to a temperature stress (V T) showed elevated levels of PCV2 DNA in all tissues. In contrast, the amount of PCV2 detected in non-infected controls was negligible for all tissues tested. Additionally, PCV2 DNA copy numbers detected in pigs exposed to PCV2 alone (V) remained low for every tissue tested. Similar to the results obtained with serum samples, none of the differences reached the level of significance due to a large variation in PCV2 copy number between pigs in the same group.

## 4 Discussion

In a recent study performed by our group, we identified stocking density and temperature as farm level risk factors linked with the severity of PMWS occurrence (Alarcon et al., 2011a). Therefore, the present study assessed whether these “environmental stressors” directly impact on PCV2 viral load, and thus could be used to develop a PMWS model which does not rely on the use of gnotobiotic pigs. To our knowledge, this study evaluates for the first time, the roles of these specific environmental stressors likely to be encountered in the field, on PCV2 replication under experimental conditions. We demonstrated that subjecting PCV2 infected pigs to environmental stress led to decreased body weight (Fig. 1) and ADG (Fig. 2) which was associated with an increased feed conversion ratio (Table 1). Furthermore, the negative effects on production parameters observed by subjecting pigs to environmental stressors were associated with increased levels of PCV2 DNA detected in the serum (Fig. 3), as well as the mesenteric lymph node, inguinal lymph node, lung and bone marrow (Fig. 4A–D).

There is overwhelming evidence that infection with PCV2 does not necessarily lead to PCVAD but can persist as sub-clinical infection. Recent research suggested a variety of co-factors, including other pathogens that are necessary to induce clinical signs of PCVAD (Opriessnig and Halbur, 2012). This phenomenon would mirror other veterinary diseases such as *Staphylococcus aureus* infection in pigs where it has been demonstrated that infection of pigs with Influenza A virus greatly increases the pathology to secondary *S. aureus* colonization in the respiratory tract (Smith et al., 2011). However, in the case of PCV2, co-infection with a secondary pathogen does not necessarily trigger the onset of PCVAD (Alarcon et al., 2011b). More recently, research has sought to determine the involvement of environmental co-factors which can potentially lead to signs of disease in PCV2 infected pigs (Alarcon et al., 2011b). Indeed, it is well established that sub-optimal husbandry conditions negatively impact on growth performance, food conversation rates, and general thriving of pigs (Prunier et al., 2010). This effect seems to be mediated through a constant elevated production of stress hormones, such as cortisol (Marco-Ramell et al., 2011; O’Connor et al., 2010). High cortisol levels have been shown to induce immune-suppression, leading to increased susceptibility to infection in different vertebrate species including animals and humans (Kick et al., 2012; Merlot et al., 2013). Furthermore, high growth performances of current commercial pig lines are suspected to favour the development of mild chronic stress, increasing disease susceptibility (Prunier et al., 2010; Rutherford et al., 2006). Overall, the changes observed in our treatment groups are in-line with earlier reported impacts of temperature and stocking density on ADG (Huynh et al., 2005; Kerr et al., 2005).

Pigs in the C group had better growth rates (ADG) for the duration of the study compared with all other treatment groups with mean ADG followed in order by V, V SD, V T and V SD T group with the lowest. Significant differences were seen in ADG from week 3 until week 8 post inoculation which coincides with detectable viral loads, possibly reflecting the impact of viraemia which peaked at day 21. At week 3 and 6 post inoculation, mean ADG was statistically significantly lower in all groups that received PCV2 compared with the C group. Also, FCR during week 6 of the study were higher in all groups compared with the C group, attributed mainly to low weight gain. The highest FCR in week 6 was seen in the V SD T group which was over 4 times the value of the V pigs. Groups V T and V SD T had similar mean ADG values at week 3, 4 and 7, which were lower than the V and C groups at week 3, 4 and 6 and only lower than the V group at week 7. A lower ADG value in V SD T than V T was seen in week 8 although this was not statistically significant. Significant differences between V T and V SD T were seen in weeks 5 and 6. A recent meta-analysis of the impact of high environmental temperature on growth rates concluded that although there was inconsistency between studies, overall there was a negative impact on growth rates (Renaudeau et al., 2011). Interestingly however, the ADG over the time-span of the experiment was further affected by the presence of PCV2, and in pigs exposed to all three stressors, virus load was highest. These data indicate a potential interaction of environmental stress and PCV2 replication. Similar to our findings, sub-optimal husbandry stress has been shown to increase susceptibility to *Mycobacteria* infection in zebrafish which was shown to be associated with elevated body levels of cortisol (Ramsay et al., 2009).

The increased replication of PCV2 observed in this study due to environmental stress being placed on the pigs can be attributed to a few factors. Firstly, pigs kept in high stocking density are more likely to come into contact with any infected pigs already in the pen and therefore are more regularly exposed to virus. Secondly, increased stress on the pig as an individual can lead to increase in serum levels of the stress hormone cortisol. This increase in serum cortisol levels has been demonstrated in pigs in response to food and water deprivation (Parrott et al., 1989), heat stress (Becker et al., 1985), social stress (Parrott and Misson, 1989a) and shipping of animals (McGlone et al., 1993; Nyberg et al., 1988; Parrott and Misson, 1989b). Higher levels of serum cortisol associated with a higher level of stress have been demonstrated to be associated with a reduced natural killer (NK) cell cytotoxicity as well as a reduced number of circulating lymphocytes in the blood (McGlone et al., 1993). As NK cells are an essential component of the innate immune system and are heavily involved in the early response to viral infection, the increased PCV2 replication observed in stressed pigs in this study may be explained by a similar inhibition in NK cell function. Further study into the cortisol levels and NK cell function in the system described in this study would indeed be interesting and could potentially further develop the PCV2 infection model.

Results from the present study showed that on average, PCV2 copies in the mesenteric lymph node, lung and bone marrow are higher in pigs from the V SD T group compared with those subjected to only one environmental stress or C pigs. In the inguinal lymph node however, it was observed that the average PCV2 copy number was highest in the V SD group. This high average was a result of one animal having a very high PCV2 copy number in this tissue. Although the daily behaviour of the pigs was not monitored, such high PCV2 copy number in the inguinal lymph node of a single pig may be a result of that pig being submissive. McGlone et al. found increased levels of serum cortisol and an associated decrease in NK activity in pen mates that were determined submissive compared with those that were determined dominant (McGlone et al., 1993).

In conclusion, inoculation with virus reduced ADG in all treatment groups at the time of peak viraemia, and in combination with high stocking density and high temperatures exacerbated the effect. Additionally, environmental stress on the animal seems to cumulatively increase viral DNA replication, which may lead to more viral particles in the tissues tested here. In essence, it seems that we have reproduced PCVAD in pigs without the need for a secondary pathogen. That being said, the large variation in viral copy number found in tissues of pigs from the same treatment group indicates that the immune response of each individual animal may play a role in the outcome of PCV2 infection. The results presented here further strengthen the case for housing pigs in favourable conditions in order to increase production parameters and reduce susceptibility to disease.

## Figures and Tables

**Table 1:** Mean weekly feed conversion ratio for groups per room.

Week p.i	R1 (V)	R2 (V SD)	R3 (V T)	R4 (V SD T)	R5 (C)	R7 (V SD)	R8 (V SD T)	R9 (V T)	R10 (V)
1	1.13	1.04	1.54	1.08	1.04	1.41	0.83	1.13	1.30
2	1.12	1.44	0.99	1.02	1.32	0.95	0.86	1.51	0.84
3	1.97	3.74	1.78	2.30	1.35	3.02	1.79	1.92	1.77
4	1.59	1.29	1.66	1.89	1.32	1.60	1.21	1.59	1.78
5	1.38	1.69	1.35	1.77	1.44	1.76	1.66	19.19	6.14
6	7.73	23.25	13.43	25.00	1.72	8.98	19.16	1.81	1.85
7	1.18	1.19	1.04	1.43	1.93	1.45	1.00	1.42	1.20
8	2.18	2.02	1.65	1.74	2.93	1.38	1.69	1.25	7.67

**Table 2:** Average weekly feed conversion ratio for each pig infected with PCV2b and subjected to environmental stress.

Week	Control	Virus only	Virus and SD	Virus and T	Virus, T and SD
1	0.92	1.01	1.06	1.11	0.81
2	1.32	0.98	1.20	1.25	0.94
3	1.35	1.86	3.38	1.85	2.04
4	1.31	1.68	1.44	1.63	1.55
5	1.44	5.51	1.66	4.31	1.72
6	1.72	7.06	16.02	7.69	22.36
7	71.93	1.16	1.32	1.53	1.22
8	3.08	5.83	1.97	1.71	2.04
